# A novel technique to restore access in patients with central venous occlusion using the Surfacer^®^ Inside-Out^®^ Access Catheter System

**DOI:** 10.1177/1129729820909730

**Published:** 2020-03-07

**Authors:** Tarik R Baetens, Joris I Rotmans, Rutger W van der Meer, Carla SP van Rijswijk

**Affiliations:** 1 Department of Radiology, Leiden University Medical Center, Leiden, The Netherlands; 2 Department of Nephrology, Leiden University Medical Center, Leiden, The Netherlands

**Keywords:** Haemodialysis access, central venous occlusion, chronic venous occlusion

## Abstract

Exhausted central venous access is a potentially life-threatening situation for patients dependent on haemodialysis. If standard guidewire recanalisation fails, unconventional venous access or central venous needle recanalisation can be considered but are often associated with higher rates of complications and/or dysfunction. Here, we report about two patients treated successfully with the Surfacer^®^ Inside-Out^®^ Access Catheter System (Bluegrass Vascular Technologies, San Antonio, TX, USA) to achieve transmediastinal central venous access.

## Introduction

Although associated with a high risk of infection and thrombosis,^1^ central venous catheters (CVCs) are frequently needed as vascular access in patients who need to start haemodialysis (HD) urgently, and in patients who are not eligible for arteriovenous access. Thoracic central venous obstruction (TCVO) can severely restrict CVC implantation causing a potentially life-threatening condition for HD patients depended on a CVC. The Surfacer^®^ Inside-Out^®^ Access Catheter System (Bluegrass Vascular Technologies, San Antonio, TX, USA) is a CE marked device which can be used to restore and preserve central venous access in patients with chronic central venous occlusive disease. Here, we report the first two patients treated with this system in the Netherlands.

## Consent

Approval by the institutional review board is not required for anonymised reports of individual cases in our institution. Both patients gave formal informed consent to use the Surfacer system to achieve HD access and gave written informed consent to use their anonymised data for this publication.

## Technique

Sedation together with local anaesthesia or general anaesthesia is advised because lack of movement is crucial while performing transmediastinal needle recanalisation and the potential painful nature of the procedure.

**Figure 1.**
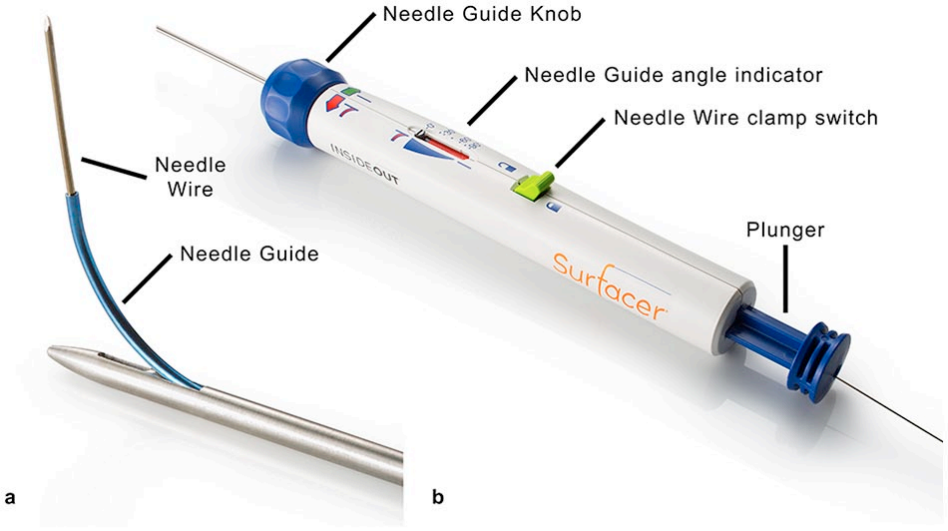
Surfacer device: (a) tip of steel shaft with needle guide and needle wire and (b) handle with needle wire.

The Surfacer system includes a long 8F sheath with dilator, two radiopaque exit targets, a 20-cm-long 16F peel-away sheath and the Surfacer device, consisting of a handle with pumping system attached to a steel shaft of length 95 cm with incorporated needle guide in the tip and a pre-inserted 180-cm-long needle wire (Figure 1).

**Figure 2.**
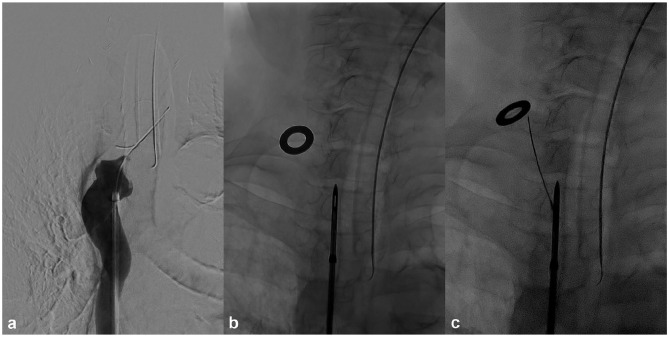
Phlebography and fluoroscopic image case 1: (a) included sheath is advanced to the venous occlusion, (b) the Surfacer device is advanced in the occluded brachiocephalic vein until the tip is just cranial of the clavicle in the anterior–posterior projection and (c) protrusion of the needle wire through the skin exiting beneath the exit target.

A short 10F sheath is inserted in the right common femoral vein by ultrasound guidance followed by catheterisation of the superior vena cava (SVC) via the inferior vena cava (IVC). Next, the included long sheath is advanced to the venous occlusion (Figure 2(a)) over a stiff Terumo wire followed by placement of the exit target on the skin above the sternal end of the clavicle. The Surfacer device is then inserted into the sheath and advanced out of the sheath under fluoroscopy into the occluded brachiocephalic vein (BCV) until the tip of the device is just cranial of the clavicle in the anterior–posterior projection (Figure 2(b)). Next, the tip and the exit target are aligned under fluoroscopy to obtain the desired degree of cranial angulation for the needle guide followed by maximising the opening in the tip by rotation of the handle to achieve proper direction of the needle guide. The needle guide is then advanced until the angle indicator matches the recorded cranial angulation followed by unlocking and advancing the needle wire through the skin by pumping the handle. Subsequently, the 20-cm long 16F peel-away sheath is introduced over the needle wire with a clamp attached just above the sheath, followed by locking the wire again and retracting the needle guide. The procedure is completed by pulling in the peel-away sheath through the handle located in the right groin and standard placement of a CVC.

### Case 1

A 70-year-old female with end-stage renal disease since 32 years due to chronic pyelonephritis. She has a access history of arteriovenous fistula (AVF) in four limbs, two lost kidney transplants, failed peritoneal dialysis after repeated peritonitis, arteriovenous graft (AVG) in both legs and anterior chest wall, placement of a Haemodialysis Reliable Outflow (HeRO) graft (Merit Medical Systems, Inc., South Jordan, UT, USA) and multiple tunnelled dialysis catheters of all four limbs. All these AVFs, AVGs and catheters were lost due to occlusions and/or chronic infection. Ultimately, the patient developed severe stenosis in the left subclavian vein (SCV), occlusion of both internal jugular veins (IJVs) and BCVs. Attempted standard guidewire recanalisation was not successful. The Surfacer procedure was performed under general anaesthesia. Instruction for use (IFU) was followed and the needle wire was easily advanced in one attempt through the mediastinal tissue and exited the skin beneath the exit target (Figure 2(c)). A peel-away sheath was pulled in over the wire, followed by uncomplicated placement of a 14.5 Fr × 24 cm Hemo-flow (Medcomp, Harleysville, PA, USA) double-lumen HD catheter. The procedure time was 35 min. Dose area product (DAP) was 9,787 mGycm^2^. Twenty-five months after the procedure, the same HD catheter still works well, without any episode of infection or dysfunction.

### Case 2

**Figure 3.**
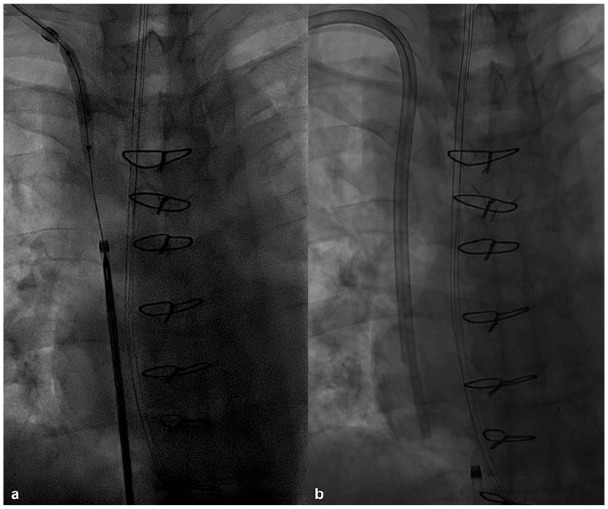
Fluoroscopic images case 2: (a) predilatation with a 6 mm × 40 mm angioplasty balloon and (b) completion image with 14.5 Fr × 24 cm Hemo-flow double-lumen haemodialysis catheter in place.

A 36-year-old male with end-stage diabetic renal failure requiring HD while waiting for approval for a kidney–pancreas transplantation. HD was stopped due to SVC syndrome caused by infected thrombotic occlusion of both BCVs. Peritoneal dialysis was started and abandoned after 2 years because of multidrug resistant pseudomonas infection requiring repeated exchange of the peritoneal dialysis catheter. Phlebography confirmed occlusion of both BCVs and collateral filling of the SVC. The Surfacer procedure was performed under deep sedation with propofol and additional local anaesthesia. The delivery system was partly advanced into the right occluded BCV followed by fluoroscopic needle guide alignment. This time advancing the needle wire was more difficult with some deviation of the wire exiting the skin cranial of the metallic ring. Pulling in the peel-away sheath over the needle wire was not successful. Despite two more punctures with slightly different skin exit areas, it was still impossible to pull in the peel-away sheath. After predilatation with a 6-mm angioplasty balloon (Figure 3(a)) and exchanging the needle wire for a Lunderquist wire, the peel-away sheath was successfully pulled in, followed by correct placement of a 14.5 Fr × 24 cm Hemo-flow (Medcomp, Harleysville, PA, USA) double-lumen HD catheter (Figure 3(b)). The difficulty of pulling in the peel-away sheath is most likely related to postsurgical changes after median sternotomy because of pericardiectomy for constrictive pericarditis 2 years before. The procedure time was 60 min. DAP was 30,007 mGycm^2^. Twenty-two months after the procedure the same HD catheter still works well.

## Discussion

HD patients with exhausted central venous access are a real clinical challenge. Occluded conventional access sites should be preserved as long as possible by standard guidewire recanalisation, if necessary with additional angioplasty and stenting. In case of failed recanalisation, two options can be considered: unconventional venous access and needle recanalisation. Unconventional percutaneous endovascular techniques are vena cava inferior access through translumbar,^2^ transhepatic^3^ and transrenal approach.^4^ These unconventional venous access routes are often poorly tolerated, technically demanding and associated with higher rates of complications and/or dysfunction^5,6^ compared with conventional access sites. Needle recanalisation was first described in 1998 by Gupta et al.^7^ and by Farrell et al.^8^ in 1999. With this technique, an occluded venous segment can be traversed with a needle, thereby establishing a connection between a peripheral vein and collateral with the central venous system. Potentially high-risk complications are haemorrhagic pericardial effusion with cardiac tamponade, mediastinal haemorrhage and arterial injury, haemothorax and pneumothorax. Literature is scarce, with case reports and small series, with the latest and largest series on central venous needle recanalisation by Cohen et al.^9^ reporting a 95% success rate in 39 patients with 5% major complications due to haemorrhage which were adequately treated with stent graft placement. This technique would only be applicable in case of a patent SCV or IJV or patent nearby collaterals. A solution would be direct transmediastinal access of the patent SVC as described by Wellons et al.^10^ in a series of 22 patients. With a standard catheter placed in the most superior portion of the SVC as guidance, the SVC was accessed from the right supraclavicular area with an 18-gauge needle under fluoroscopic guidance. The procedure was successful in all patients but complicated with pneumothorax in two patients. Transmediastinal inside–out central venous access (IOCVA) was first described by Elayi et al.^11^ in a case series of eight patients with central venous occlusion requiring lead placement for rhythm management devices. Access was achieved without significant complications with the use of a transseptal sheath set with a modified transseptal needle to guide a 0.018″ needle wire for the inside–out puncture. They describe a consistent anatomic relationship on basis of gross anatomy where the central veins are anteriorly bounded by soft tissue, clavicle and skin providing a safe needle trajectory whereas arteries, nerves and veins lie posteriorly and lateral of the central veins. Several modifications to this technique have been recently reported. Hadziomerovic et al.^12^ use a modified technique in nine patients with a manually shaped 80-cm metal stiffening cannula from a percutaneous gastrojejunostomy catheter kit together with a guidewire to perform a blunt mediastinal dissection followed by deployment of a snare loop in the subcutaneous tissue. After ultrasound examination of the proposed tract on possible arteries, percutaneous fluoroscopic puncture was performed through the snare to gain a through-and-through wire. They report a large hemothorax as the only intraprocedural complication and 100% technical success. Freeman et al.^13^ report a slightly modified technique by using a long braided transseptal sheath without a transseptal needle, to guide the inside–out puncture in eight patients. Access was achieved in all patients without complications. Murga et al.^14^ combine the inside–out approach with a HeRO graft to achieve arteriovenous access in three patients.

The Surfacer system was developed based on the IOCVA technique to provide solely right-sided supraclavicular transmediastinal needle recanalisation. The steel shaft of the system has been made fairly rigid to ensure that it can be pushed in a straight line through the occlusion(s) to the supraclavicular area. Because of this rigidity, excessive spinal curvature, vascular tortuosity, vascular anomaly including aneurysmatic disease of the ascending aortic arch and the brachiocephalic trunk are relative contraindications which could lead to inability to advance the device or lead to an altered course of the device with high risk complications similar to central venous needle recanalisation. Because of these risks, left femoral access with known oblique course of the common iliac vein and steep angle with the IVC is contraindicated for use. Other contraindications for use are occlusion of the right femoral vein, iliac vein or IVC or acute thrombosis in a vessel to be crossed by the Surfacer system. A contrast enhanced pre-procedural computed tomography (CT) venography is mandatory in identifying the contraindications. The Society of Interventional Radiology (SIR) has published reporting standards for TCVO, identifying different types of obstruction.^15^ The Surfacer can be used in type 1 (occlusion of the right IJV), type 2 (occlusion of the right BCV), type 3 (bilateral occlusion of the BCV including the confluence above the azygos) and type 4 (occlusion of the SVC between the azygos vein and the right atrium) obstructions. Type 4 TCVO is only treatable with a visible short stump of the SVC above the right atrium. This stump is mandatory to identify the entrance of the SVC and so providing a safe route for the wire and device out of the right atrium and avoiding atrial perforation.

First experience with the device is described in the *Endovascular Today* journal as part of a safety and feasibility study with 12 successfully treated patients at the Italian Hospital in Asuncion, Paraguay.^16^ In 2016, CE mark certification was received followed with commercial availability outside the United States.

In October 2018, enrolment has closed for the ‘Surfacer System to Facilitate Access in Venous Occlusion’ (SAVE) registry, a post-market, prospective, single-arm, multi-centre, international European registry to evaluate the clinical outcomes of 30 patients followed by the start of the SAVEUS (Surfacer System to Facilitate Access in Venous Occlusions – United States) trial, a pre-market investigational device exemption study to evaluate the safety and efficacy in the United States. Definitive results of the SAVE registry are still pending. In our limited experience, the Surfacer Inside-Out Access Catheter System provides a good alternative to restore venous access in patients with chronically occluded veins where standard guidewire recanalisation has failed. In both patients, venous access for HD was restored with long-term patency without complications or adverse events.
